# Greatest Hits—Innovative Technologies for High Throughput Identification of Bispecific Antibodies

**DOI:** 10.3390/ijms21186551

**Published:** 2020-09-08

**Authors:** Tim Hofmann, Simon Krah, Carolin Sellmann, Stefan Zielonka, Achim Doerner

**Affiliations:** 1 Advanced Cell Culture Technologies, Merck Life Sciences KGaA, Frankfurter Strasse 250, D-64293 Darmstadt, Germany; tim.hofmann@merckgroup.com; 2 Protein Engineering and Antibody Technologies, Merck Healthcare KGaA, Frankfurter Strasse 250, D-64293 Darmstadt, Germany; simon.krah@merckgroup.com (S.K.); carolin.sellmann@merckgroup.com (C.S.); stefan.zielonka@merckgroup.com (S.Z.)

**Keywords:** bispecific antibodies, high throughput screening, protein trans splicing, inteins, DuoBodies, controlled Fab-arm exchange, Sortase A, microbial transglutaminase, SpyTag, SpyCatcher

## Abstract

Recent years have shown a tremendous increase and diversification in antibody-based therapeutics with advances in production techniques and formats. The plethora of currently investigated bi- to multi-specific antibody architectures can be harnessed to elicit a broad variety of specific modes of actions in oncology and immunology, spanning from enhanced selectivity to effector cell recruitment, all of which cannot be addressed by monospecific antibodies. Despite continuously growing efforts and methodologies, the identification of an optimal bispecific antibody as the best possible combination of two parental monospecific binders, however, remains challenging, due to tedious cloning and production, often resulting in undesired extended development times and increased expenses. Although automated high throughput screening approaches have matured for pharmaceutical small molecule development, it was only recently that protein bioconjugation technologies have been developed for the facile generation of bispecific antibodies in a ‘plug and play’ manner. In this review, we provide an overview of the most relevant methodologies for bispecific screening purposes—the DuoBody concept, paired light chain single cell production approaches, Sortase A and Transglutaminase, the SpyTag/SpyCatcher system, and inteins—and elaborate on the benefits as well as drawbacks of the different technologies.

## 1. Introduction

The human body is continuously exposed to potentially harmful and life-threatening opponents such as pathogens or malignant cells. In order to assert oneself, several layers of innate defense mechanisms such as pathogen recognition receptors enable the early detection of ‘danger signals’ and, consequently, the elimination of the respective invader [1,2]. Moreover, the human immune system can also respond in an adaptive fashion to a foreign antigen via antibody-mediated processes, for instance antibody-dependent cellular cytotoxicity (ADCC) or complement-dependent cytotoxicity (CDC) [3,4]. As such, antibodies are crucial players in host defense. Inspired by the natural mechanisms of antibody-mediated pathogen elimination, monoclonal antibodies emerged as one of the most promising classes of therapeutic molecules, with about 80 entities now being approved either in the USA or Europe [5,6]. However, one of the main limitations of antibody-derived therapeutics relies in their monospecific nature. This restricts targeting to one antigen only. Since most diseases are typically rather complex, involving multiple disease mediators or factors simultaneously [7,8], much effort was made within the last decades to combat the multifaceted nature of oncology and immunology diseases more efficiently. In this respect, the advent of bispecific antibodies opened up new avenues for disease treatment such as effector cell redirection or the simultaneous blocking of two different disease mediators, with encouraging results in (mostly early stage) clinical trials [9,10,11]. Consequently, an unprecedented multitude of bi- and multi-specific formats employing flexible valences and overall architectures were engineered to facilitate a plethora of modes of action (MoA), as elegantly described elsewhere [9,12].

For the generation of bispecific antibodies, one of the main tasks relies in the identification of paratope pairs that fulfill the desired MoA in the best way possible when reformatted as bispecific. Nowadays, antibody selection campaigns such as immunization combined with B-cell cloning, Phage Display, or Yeast Surface Display of naïve, synthetic, as well as immune libraries typically enable the identification of up to hundreds of different initial hit candidates [13,14,15,16,17]. Hence, testing of every combination of hits for each binding arm in a bispecific antibody format would result in the expression of several thousands of different molecules, making the whole process rather cumbersome and tedious. In this regard, Kitazawa and co-workers initially expressed and analyzed 40,000 bispecific antibody combinations in order to identify a molecule that mimics the cofactor function of coagulation factor VIII sufficiently [18]. In addition, other factors such as the order of paratopes in a given architecture or steric hindrance might impact biological activities, e.g., crosslinking abilities for effector cell redirection, and thus need to be taken into consideration [19,20,21]. Consequently, full coverage of this multidimensional screening space would require huge efforts with respect to molecular biology, i.e., cloning, antibody expression, and purification.

**Figure 1:**
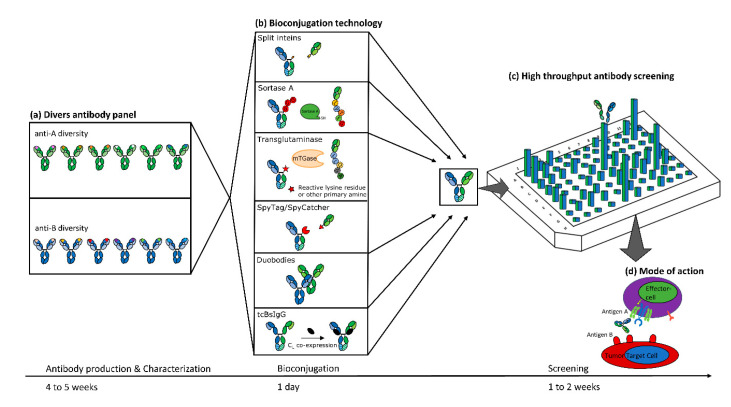
Bispecific antibody generation using different bioconjugation technologies with subsequent high throughput screening and characterization. (**a**) A diverse panel of up to hundreds of anti-A and anti-B paratopes are generated and characterized within 4 to 5 weeks. (**b**) A bioconjugation technology is selected to generate a bispecific IgG full-length format with correct chain pairing. For the sake of simplicity in this example, Fab fragments are reconstituted with one-armed monovalent fragments. The depicted antibody fragments contain a heterodimerization technology indicated by striped CH_3_ regions. The bispecific antibody reconstitution is performed in a combinatorial setup to increase the number of variants. (**c**) High throughput antibody screening of reconstituted bispecific antibodies is performed in plate format, depending on the mode of action. (**d**) Biological functional screening of several T-cell engager combinations is conducted in 1 to 2 weeks, depending on the combinatorial sample size.

In order to overcome this bottleneck, technologies such as controlled Fab-arm exchange used for DuoBodies [22], paired light chain single cell production approaches [23], microbial transglutaminase [24] or Sortase A [25] mediated bioconjugation, the SpyTag/SpyCatcher system [26,27], or split inteins [28] were adapted to enable broad bispecific antibody screening (Figure 1). These methodologies harbor the benefit that bispecific entities can be generated from a pre-existing set of antibody fragments in a mix-and-match manner, significantly reducing hands-on time as well as overall efforts for bispecific screening. In this review, we aim at giving an overview of the most relevant protein bioconjugation technologies for the creation of bifunctional antibodies. We further discuss benefits as well as limitations of each specific methodology in the context of bispecific screening.

## 2. Controlled Fab-Arm Exchange (“DuoBodies”)

A very elegant and generic approach for the generation of bispecific antibodies was adapted from processes occurring within the human immune repertoire itself. IgG4 antibodies are dynamic molecules that physiologically form bispecifics by a process called Fab-arm exchange (FAE). Herein, half molecules (consisting of a paired heavy and light chain) from different IgG4s recombine and form a polyclonal mixture of bispecific antibodies in vivo. By losing their ability to crosslink antigens and generate immune complexes, it is believed that IgG4 may dampen inflammatory reactions by interfering with immune complex formation of other antibody isotypes in chronic inflammatory diseases [29]. The natural mechanism of FAE also seems to be attractive to generate bispecific antibodies in vitro, because it may overcome limitations of other bispecific antibody platforms like Knobs-into-Holes [30], wherein additional methods like CrossMab [31] or paratope scFv grafting need to be applied to circumvent light chain mispairing [7,32].

In 2013, Genmab published an “Efficient generation of stable bispecific IgG1 by controlled Fab-arm exchange” [22]. This work was inspired by the observation that human/rhesus intraspecies IgG4 enhance FAE (due to IgG4 specific CH3 residues) and that 2-mercaptoethylamine-HCL (2-MEA), as a mild reducing agent, enables FAE with antibody pairs harboring native IgG1-hinge regions. In controlled Fab-arm exchange ((c)FAE), two IgG1s with matched CH3 mutations are produced separately and are subsequently recombined under reducing conditions. CH3 mutations need to destabilize the CH3 homodimer interaction and promote heterodimer formation. For this, the IgG4 specific R409 mutation was incorporated into one IgG1 CH3 and combined with surrounding mutations on positions L368, K370, D399, F405 and Y407 (all amino acids except for C and P) on the opposite chain. In a dual-binding ELISA, matched point mutations for all five antibody positions were obtained, enabling bispecific binding by FAE. In depth analyses of (c)FAE were conducted using IgG1-F405L and IgG1-K409R mutants, the variants that are also applied in Genmab’s proprietary DuoBody technology. The general process of bispecific antibody generation applying DuoBodies involves three basic steps: (1) separate production of monospecific antibodies harboring respective mutations in mammalian cell cultures, (2) purification according to standard processes (e.g., Protein-A), (3) (c)FAE under tailored laboratory conditions followed by another purification step. This typically yields bispecific antibodies with more than 95% heterodimer content. The beauty of DuoBodies lies in the fact that the bispecifics fully retain IgG1 structure and that Fc-mediated mechanisms, such as FcRn mediated recycling and Fcγ-receptor interactions, stay unaffected [22]. Today, several bispecifics based on (c)FAE are under clinical development. One of these molecules is the MET × EGFR bispecific antibody JNJ-61186372, jointly developed by Genmab and Janssen, currently in Phase I, for the treatment of advanced NSCLC, that was screened from five anti-MET × 8 anti-EGFR parental antibodies in the DuoBody format [33].

Similar to the bioconjugation methods described in later sections, (c)FAE serves as an ideal platform for bispecific antibody screening. For the given example of 40,000 antibody variants individually produced and screened by Kitazawa et al. [18], only 200 + 200 = 400 parental monospecific would need to be produced using complementary DuoBody mutations, while 200 × 200 = 40,000 variants could be screened, drastically reducing lab work and timelines applied during classical screening efforts [7,32].

Associated with that, AstraZeneca recently published a method for high throughput bispecific antibody screening, applying matched F405L and K40R mutations [34]. In addition, H435R and Y436F (also known as RF-mutation) were incorporated into one of the CH_3_ mutants (K409R). The RF-mutation completely abrogates the interaction of an antibody-Fc with Protein-A (however, it should be noted, that antibodies harboring a VH3 segment will bind to Protein-A, regardless of the RF-mutation in the antibody-CH_3_). In a proof of concept study, bispecific antibodies were generated from four parental antibodies (anti-Her2; anti-EGFR; anti-NIP228; anti-IGF1R). All six resulting bispecific antibodies could be produced with high heterodimer purity as demonstrated by liquid chromatography-mass spectrometry (LCMS) and reverse phase liquid chromatography (RPLC) and showed bispecific target engagement proven by BLI. Compared to the conventional DuoBody generation, this approach enables the formation of bispecifics directly from culture-supernatants without any pre-purification steps of monospecific antibodies, making it perfectly suited as a bispecific screening tool [34].

Finally, bispecific antibodies can be generated by the DuoHexaBody platform, developed by Genmab, combining the before mentioned DuoBodies with a hexameric formation of antibody complexes. The DuoHexaBody technology offers a broad applicability for bispecific antibody generation with target-mediated enhanced potencies for CDC-mediated effector functions as elegantly demonstrated by Oostindie et al. [35].

## 3. Paired Light Chain Single Cell Production Approaches

When aiming at screening of heterodimeric Fc-based bispecific antibodies not affected by Light Chain (LC) pairing issues, the combination of two binding moieties’ panels can also be executed on the DNA rather than the protein level. Genentech developed a tethered-variable CL bispecific IgG (tcBsIgG) platform based on Knobs-into-Holes heterodimerization combined with linked light chains. This approach allows robust production of intact bispecific antibodies in a single cell line, concurrently ensuring cognate light chain pairing and preserving structural and functional properties of heterodimeric Fc antibodies, hence rendering it applicable to high throughput screenings [36]. Another elegant example is a combination of a common LC with a DEKK heterodimerization Fc portion single cell production of 545 bispecific antibodies for a successful unbiased HER2/HER3 combinatorial screen at Merus [23].

In addition to methodologies for screening of classical IgG and heterodimeric Fc architectures, plentiful approved or clinically-evaluated non-Fc formats represent a need for further high throughput screening technologies beyond Fc heterodimerization strategies. Enzyme-mediated bioconjugation, up to now mainly applied for ADC generation, could be a solution.

## 4. Microbial Transglutaminase and Sortase A

Enzyme-mediated bioconjugation has evolved in the last decades to a robust and versatile tool for protein labeling and covalent attachment of two or more protein building blocks by site-specific ligation. It also obviates the need for harmful chemicals as employed for chemical ligation. Finally, also enzymatic bioconjugation avoids cumbersome genetic engineering or subsequent expression of individual multivalent binders but offers options for one-pot reactions yielding bispecific antibodies amendable to high throughput or functional screening approaches.

First clues for the presence of transglutaminases (TGases) date back to the work of Clarke and colleagues in 1957 describing the transamidation activity by pig liver [37]. Microbial transglutaminases (mTGases) were first isolated from *Streptomyces mobaraensis* [38] and are now widely used in the food, textile, and cosmetic industry as “biological glue”, e.g., for meat [39]. Belonging to the class of protein-glutamine γ-glutamyltransferases, TGases catalyze the transfer of primary amines (e.g., the ε-amino group of lysines as acyl-acceptor) to γ-carboxamides (e.g., γ-glutamyl group of glutamine as acyl-donor) under ammonia release, yielding a stable isopeptide bond resistant to proteolytic degradation [39]. Although mTGases accept a promiscuous acyl-acceptor substrate repertoire [40], surface-exposed glutamines of glycosylated native human IgG1 antibodies are not accepted by mTGase [41]. mTGase has been engineered by directed evolution and rational design for improved catalytic performance and alternative recognition sequences [42,43], and has been exploited in particular for the generation of antibody-drug conjugates [41,43,44] or labeling of antibody fragments [45,46]. Multimerization of a single domain antibody fragment targeting TNF could be achieved by using mTGase and compatible peptidyl linkers [24]—an approach that could be generically employed for high throughput screening of non-Fc bispecific antibodies.

The *Staphylococcus aureus* transpeptidase Sortase A (SrtA) was identified in 1999 to be essential for cell wall assembly of gram-positive bacteria, recognizing a conserved “LPXTG” motif within secreted cell surface proteins, cleaving between the threonine and the glycine residues, forming an thioester acyl-enzyme intermediate and catalyzing the covalent linkage of the carboxyl group of the threonine to a nucleophilic amino group of a N-terminal pentaglycine within the cell wall anchored peptidoglycan [47]. The reaction is reversible, hence, dependent on an excess of donor and acceptor substrate to favor high yields, with the acylation step being the rate-limiting factor [48]. Removal of unwanted byproducts can limit the reversibility of the reaction and could provide the basis for an equimolar ratio of the substrates. Sortase A engineering yielded variants with improved reaction rates and the reduction of by-products [25,49,50,51,52] with versatile applications also termed “sortagging” [53,54,55] such as protein circularization [56] and antibody drug conjugates [57].

SrtA has been employed for the generation of bi- and multi-specific antibodies of several formats, e.g., conjugations of Fab [55] or scFv fragments [58] as well as C-terminally linked antibody heterodimers in combination with click chemistry [59]. For a high throughput screening approach, the group of Plückthun recently established a one-pot SrtA-mediated coupling reaction for the alternative binding protein DARPin (designed ankyrin repeat proteins) with compatibility to functional assays, due to minimal levels of monovalent side products. Combinations of 21 DARPins, resulting in 441 bispecific molecules targeting c-MET and EpCAM, were analyzed for cell proliferation inhibition and yielded a novel bifunctional DARPin with superior cellular activity [25]. This illustrates the applicability of enzymatic bioconjugation for pharmaceutical high throughput biotherapeutics’ functional screening approaches.

Another bacterial transpeptidase served as the basis for the engineering of a ligase, for the conjugation of two proteins. This system, also known as the SpyTag/SpyCatcher system, provides a fully intrinsic bioconjugation capability without the need to add enzymes and is described below.

## 5. The SpyTag/SpyCatcher System

The SpyTag/SpyCatcher system originates from covalently-stabilized pilin polymers found in the pilus of Gram-positive bacteria which usually undergo intramolecular amide bond formation to impart mechanical and proteolytic stability to pili [60], precisely *Streptococcus pyogenes*, inspiring the name Spy Technology. When split and engineered, Zakeri and co-workers generated first generation isopeptags binding to pilin-N or -C [61,62], mediation of irreversible peptide-peptide interactions via SpyLigase [63], and finally, the SpyCatcher and SpyTag [64]. This approach makes use of the collagen adhesin domain (CnaB2) of fibronectin binding protein (FbaB) that possesses an internal isopeptide bond between N- and C-domains’ amino acids Lys31 and Asp117. When engineered, the domain is split between Lys and Asp into two fragments resulting in an N-terminal fragment (SpyCatcher) of 138 aa and a C-terminal fragment (SpyTag) of 13 aa. Putting both fragments again in close proximity, a spontaneous formation of a covalent peptide bond between Lys and Asp occurs, resulting in a double hydrogen bond between Glu77 and Asp117, facilitating the peptide bond formation by nucleophilic attack and forming a zwitterionic intermediate. The bioconjugation of SpyTag and SpyCatcher is redox insensitive, efficient in a broad range of pH (5 to 8) and temperatures (4 to 37 °C) [62]. The ligation reaction rate is fast and was further increased 12-fold by engineering an optimized SpyCatcher002 variant yielding complete bioconjugation after a few minutes [65].

The technology is a promising tool for diverse biotechnological applications as reviewed by Sutherland et al. [26], as discussed also above, ranging from protein ligation via peptide bond formation to protein stabilization by cyclization [65,66,67]. Specifically, the SpyTag/SpyCatcher fragments can either be fused C- or N-terminally or to internal positions within the protein, unlike, for example, split inteins, which are restricted to C- or N-terminal fusions. Within the years, a plethora of applications has been published, ranging from the generation of bispecific antibodies to ROBO1 [68], a generic trivalent scFv platform [69], and the generation of site-specific conjugated ADCs with high efficiency [70] for therapeutic approaches. Akiba et al. described an elegant technology for generating biparatopic antibodies through two-step targeting using a pair antibody-SpyTag or SpyCatcher-fusions targeting different epitopes of the same target that spontaneously react to form a covalent bond between fragments to generate a biparatopic antibody in situ [71]. The system is applied in several modular “plug-and-display” approaches for the generation of enhanced immunostimulants against cancer or infectious diseases such as influenza or MERS-CoV [72,73,74,75,76]. Further applications include optimized protein purification procedures (in parts, in combination with inteins) [77,78], specific immobilization of targets for enhanced phage display antibody discovery campaigns [79], site-specific fluorescence labeling of antibodies for in vivo optical imaging [80], up to in vivo assembling of proteins or enzymes [81,82], and versatile further applications, in depth reviewed by Hatlem et al. [27].

In addition to a two-component Spy system, Veggiani and co-workers identified an orthogonally acting SnoopTag/SnoopCatcher pair by engineering *Streptococcus pneumoniae* adhesin RrgA in a similar fashion, which also forms a spontaneous isopeptide bond with >99% yield and no cross-reaction to SpyTag/SpyCatcher [83]. Similar to Spy approaches, three-part splitting of RrgA resulted in the respectively called SnoopLigase, catalyzing the formation of an isopeptide bond between the two peptide tags, SnoopTagJr and DogTag [84].

Many biotherapeutics currently in pre-clinical research and early development are complex molecules, mostly bispecific antibodies, and require both a specific spatial arrangement and flexibility of their binding moieties to not only specifically bind but also fully elicit their functionality of often several desired MoAs [85,86]. Therefore, in-format screening of bispecific antibodies is desirable and can significantly impact the identification of the best binders’ combination [87]. SpyTag/SpyCatcher fusions leave a peptide imprint of 151 aa to the protein of interest after bioconjugation. A 32 aa shortened, truncated version of an N-terminal SpyCatcher fragment has been developed by Li et al. [88] to decrease an immune response in mice and to shorten the peptide footprint. In case of screening for effector cell engagers, the remaining 13 kDa Spy domain could lead to altered flexibility and paratope distances, to impaired biological functionality, and finally, to potentially suboptimal choice of binders or epitopes. Despite these potential limitations for screening of certain biotherapeutics classes, the SpyTag/SpyCatcher system has, only ten years after its initial publication, been applied to a tremendous extent and very versatile manner and will continue to drive basic science as well as pharmaceutical development.

## 6. Split Inteins

Another option for in vitro bioconjugation and screening of bispecific antibodies are split inteins. Found in every organism of life, N- and C-termini of split inteins are located in their natural origin on two different genes to elicit splicing in trans at the protein level with well-understood mechanisms [89]. The process is called protein trans splicing (PTS), and it yields—similar to enzymatic bioconjugation or Spy technology—a stable peptide bond and is very specific for each split intein [90]. PTS is a single irreversible turnover reaction, not dependent on thermal or energetic co-factors such as temperature or ATP. The most common and well investigated split inteins are *Ssp* DnaB from *Cyanobacterium synechocystis* [91] or *Npu* DnaE originated from *Nostoc punctiforme* with a reported splicing rate of t_1/2_ = 1 min at 37 °C [92,93,94]. Most investigated split inteins are dependent on a mild reducing environment by the addition of reducing agents like Tris (2-carboxyethyl) phosphine (TCEP) or dithiothreitol (DTT) to undergo PTS.

PTS is dependent on intein and extein sequences. Exteins flank both split intein parts holding specific recognition sequences to initiate the PTS mechanism. The amino acid residue at position +1 consists either of a cysteine, serine, or threonine, depending on the split intein. PTS takes place in four steps: (1) a nucleophilic attack by the first amino acid residue of either a cysteine or serine located in the N-terminal intein part on the carbonyl carbon of the preceding amino acid residue (−1 position) located in the flanking N-extein leads to an N-S or N-O acyl shift; (2) a linear (thio) ester intermediate being trans-esterificated by a nucleophilic attack of the first amino acid residue of the so-called C-extein (+1 position) forms a branched intermediate [95]; (3) the N-terminal intein part is now cleaved from its fusion protein and transferred to the N-extein. As a result, a succinimide ring is generated through cyclization of the conserved asparagine residue of the C-terminal intein part after the nucleophilic attack of the previously formed intein extein junction; (4) finally, the cleavage reaction of the C-terminal intein part followed by a spontaneous S-N or O-N acyl shift ligates the esterified C- and N-exteins by a native stable peptide bond [96,97]. The process of PTS is similar to native chemical ligation (NCL), which is well described elsewhere [98]. Similar to (c)FAE, mild reducing conditions for the activation of certain inteins have been proven to minimize the potential risk of interchain disulfide bond breaks but retain native LC pairing and the desired functionalities of resulting bispecific antibodies. Non-antibody cysteine residues within extein sequences are essential for certain inteins and could lead to disulfide scrambling or the generation of trisulfide bonds [99]. Of note, some split inteins rely on serine or threonine for catalytical activity, hence do not require cysteines and are engineered to undergo PTS triggered by a shift in pH, salinity conditions, light or temperature, making them more applicable for a variety of biological applications [92,100]. Recently, the engineered cysteine-free version of the split intein *Aes* PolB1 intein was developed by Bhagawati and coworkers, with fast kinetics and not depending on a reducing environment, enabling the screening of antibodies exhibiting disulfide bonds sensitive to reduction [101]. In addition, and similar to SpyTag/SpyCatcher, most inteins require the incorporation of a one to six extein amino acid footprint that may cause altered flexibility and geometry. To overcome this limitation, serine- or threonine-based inteins [100] could be applied for seamless bioconjugation at respective, naturally occurring sequence positions or exteins could replace amino acids within the IgG hinge region or linker sequences in further formats during screening. Finally, the incorporation of split inteins into a nonnative host protein generally could alter splicing kinetics resulting in non-ligated products [102,103], hence this needs to be individually assessed for each bispecific antibody format.

Han et al. first described in vitro bioconjugation of a full-length bispecific antibody via intein fusions to precursor antibody fragments within the hinge region and successfully demonstrated in vivo activity of a reconstituted CD3xHer2 T-cell engager mediated by *Npu* DnaE [28]. A similar approach yielded a CD3xPRLR bispecific antibody for T-cell activation and cytokine release towards PRLR expressing breast cancer cells [104]. In addition to Fc-based bispecifics, also non-Fc, circularly connected VHH fragments (cyclobody) have been developed via SICLOPPS (Split Intein Circular Ligation of Peptides and Proteins) reaction between both C- and N-termini, forming a cyclic conformation after PTS [105]. These cyclobodies are, as discussed before for other applications, protected from proteolysis due to their cyclic topology, yet they retain their dual specificity. An anti-EGFRxCD16 cyclobody was successfully generated to show cytotoxicity against EGFR-positive cancer cells, able to bind simultaneously EGFR and CD16 on the cell surface [105]. Applicability of the aforementioned split intein *Aes* PolB1 intein was demonstrated by successful bioconjugation of several therapeutically relevant formats like full-length IgG, Fc, and VHH fusions [101]. Split inteins have been used to bioconjugate toxic components to antibodies [106], avoiding toxicity issues during antibody production, as exemplified by an anti-Her2 immunotoxin conjugated via split intein derivative (M86) of the *Ssp* DnaB intein [107]. Next to several split inteins applied in bioconjugation of single binders in diverse formats, we recently described the application of split inteins for automated high throughput screenings in pharmaceutical research and development [108]. Similar to other presented methodologies, split intein screening could enable the comparison of complex formats with feasible low production needs and faster development times.

In addition to such two component systems, and akin to the Spy/Snoop combinations discussed earlier, orthogonally-acting split intein pairs could enable three to multi component screens [109], e.g., for trispecific antibodies. The group of Pinto et al. [110] presented a mutually orthogonal split intein library for in vivo applications and further split intein pairs to be used for the in vitro seamless assembly of large repetitive proteins with biotechnological or pharmaceutical relevance.

## 7. Conclusions and Outlook

The past years have seen the unparalleled development of monospecific antibodies with natural MoAs as well as a broad range of engineered bispecifics to multi-functional biologics for therapeutic intervention in cancer or immunological diseases. The concept of bispecific antibodies for effector cell recruitment dates back to the 1980s, with reports on production [111] and effective CTL retargeting [112] and the design of a universal T cell engager [113]. However, it was only until a decade ago that Catumaxomab (Revomab^®^) for targeting EpCAM-positive tumors by Fresenius Biotech as well as Micromet and Amgen with CD19-specific Blinatumomab (Blincyto^®^) were the first to bring bispecific T cell engaging antibodies to clinical approval [114]. This resulted in a tremendous increase and diversification of bispecific antibody approaches for cancer and immunotherapy in research and clinical development [5,9,12]. Many investigational bispecific antibodies still aim at retargeting T cells to kill tumor cells [115,116] of both solid and hematological malignancies, exemplified by a BiTE platform review for cancer immunotherapy [117]. A similar, flourishing strategy in cancer immunotherapy involves the recruitment and activation of NK cells, as applied in Bispecific or Trispecific Killer cell Engagers (BiKEs or TriKEs), recently reviewed for approaches towards solid [118] and hematological diseases [119]. Other MoAs aim at intervening with two different disease mediators such as cell surface receptors, soluble ligands, and other proteins [116]. Diverse architectures allow various numbers and spatial relationship between different binding sites, valences, several secondary immune functions, and pharmacokinetic half-life. This plethora provides great opportunity to tailor the design of bispecific antibodies to match the desired MoAs and the intended clinical application to address the unmet need in several cancer indications [116].

Advances have been reported in production, often by site-specific bioconjugation and self-assembly technologies, and reviewed elsewhere [120]. The sheer size of the combinatorial screening space spanning between diverse panels of targeting moieties, formats, and MoAs demands innovative methodologies for automated high throughput functional screenings of bispecific and even more complex formats in pharmaceutical development. As the plentiful formats and MoAs of the biology-driven discovery of bispecific antibodies was recently elegantly reviewed by Nie et al. [121], we sought to cover methodologies technically enabling broad bispecific screenings. Until today, the successful development of highly innovative bispecific antibodies was carried out by efficient automated screening with appropriate robotics, as exemplified by the work of Kitazawa et al. [18] or Geuijen and co-workers [23]. In addition to the technologies described herein, classical screening will continue to yield highly differentiated biotherapeutics such as bispecific antibodies with optimized MoAs. Complementary to this, most screening methodologies described herein share the opportunity to greatly reduce production needs and development times for complex biotherapeutics by bioconjugation on the protein level or when produced in a small scale from one cell. Classical drug candidate screening is often straight forward and allows for functional interrogation in the desired final format, whereas methodologies presented herein add a layer of complexity with inherent advantages and drawbacks, discussed in the following paragraph. The choice of an appropriate method will often be based on locally established automation, expertise, and the respective desired format and associated MoA to be screened.

For heterodimeric Fc architectures, (c)FAE, tethered variable-CL, or common LC bispecifics avoid light chain mispairing issues and represent viable options with minimal engineering but robust conversion, enabling high throughput screenings in early stages even in crude supernatant. Similarly, PTS can be performed both in vitro and in vivo, hence enabling bioconjugation without preceding purification within the production cell or in the supernatant [120,122]. This represents an advantage over enzymatic ligation for early bispecifics HTS. Enzyme bioconjugation, as exemplified by mTGase and SrtA, can be applied for non-Fc formats, leaving minimal peptide footprints, but requires enzyme addition and, potentially, purification. Unlike DuoBodies, the Spy technology and split inteins are not restricted to the full-length IgG antibody format and are applicable to pharmaceutical development high throughput screening of multiple formats by fusing binding moieties to the termini of a Fc portion, or to each other and also overcome light chain pairing issues during screening. Although the SpyTag/SpyCatcher system has been harnessed for versatile applications in the past years, the significant imprint of one additional protein domain in the resulting bispecific product might hinder its application for the screening of formats strongly relying on the respective paratope distance to elicit the desired MoA, e.g., effector cell recruitment. Split inteins generate a comparatively low scar of 3 to 6 amino acids remaining that, e.g., in linker sequences, can be engineered to mimic the envisioned format, representing a viable option for close-to-format or, under certain conditions, in-format screening.

Both the combination of Spy/Snoop technology and orthogonal split inteins offer the option to screen even larger combinatorial spaces of tri- to multivalent biologics, such as biparatopic effector cell engagers, bispecific, ADCs, or trispecific antibodies. In addition to antibody-derived bispecifics, the plethora of alternative scaffolds fused to relevant antibody portions might further enlarge the combinatorial space that could be covered by the discussed screening methodologies, as recently exemplified for PTS-generated, lectin-based antibody fusions termed “Lectibodies” [123]. Limitations and advantages apply similarly to the above discussed two component approaches. Table 1 gives an overview of the properties, advantages, and shortcomings of the respective methodologies. The future will tell whether a broader coverage of these large screening spaces by application of reviewed or similar technologies will enable the identification of best-in-class innovative biotherapeutics.

Although this review discussed mainly the screening of IgG-based therapeutic bispecific antibodies, as well, isotype-switched IgA, IgD, IgE, and IgM antibodies play important roles in the healthy and diseased immune system, with distinct differences in the architecture, tissue distribution, receptor binding, and effector function properties. Respective isotypes and their application for bispecific antibodies were recently comprehensively reviewed [124] and might bear great potential for future bispecific antibody therapies.

In summary, the reviewed methodologies offer great tools for broad functional screening campaigns of diverse bi- to multi-specific antibodies and formats, with versatile MoAs in early drug discovery. These methods could greatly shorten development times and enhance the probability of identifying optimal combinations, ultimately leading to the generation of better biotherapeutics.

## Figures and Tables

**Table 1:** Comparison between different bioconjugation technologies for posttranslational antibody modification. The technologies presented here are able to reconstitute antibody fragments in vitro on the protein level. Tethered variable CL as well as common LC bsAbs are generated on a molecular biology basis and not recombined on the protein level.

Technology	Linkage	Component Number	Handle of Motif	Residual Amino Acid Imprint	Activation Conditions	Reaction	Cofactors	Downstream Purification	HTS Compatibility	In Vivo Ligation	Covered Formats
Duobodies	None	2	F405 and Y407 substitution in CH3	Seamless	Mixed mAb precursor fragments under mild reducing conditions	Controlled Fab-arm exchange	Reducing agent, 2-MEA	Protein A, SEC	Yes	No	Full length-IgG format, DuoHexaBodies
Transglutaminase	C-terminal, N-terminal, site specific	2	LLQGA	LLQGA	Addition of Transglutaminase in high concentrations	Acyl-transfer	None	Protein A, SEC	No	No	Antibody drug conjugates
Sortase A	C-terminal, N-terminal, site specific	2	LPXTG	LPXTGGG	Excess of one reconstitution partner required based on a reversible reaction, high concentrations of Sortase A	Transpeptidation	None	IMAC	yes	Yes	Fc fusions, scFv, VHH
SpyTag/SpyCatcher	C-terminal, N-terminal	3	Lys in SpyCatcher, Asp in SpyTag	SpyTag/SpyCatcher	Isopeptide bond formation after bringing precursor proteins in close proximity	Amidation	None	Protein A, SEC	Yes	Yes	Full length-IgG format, Fc fusions, scFv, VHH
Split inteins	C-terminal, N-terminal	3 or more	Int ^N^ and Int ^C^	Extein sequences	Isopeptide bond formation after bringing precursor proteins in close proximity under mild reducing conditions	Protein trans splicing	Reducing agent TCEP, DTT if using cys containing split inteins	IMAC	Yes	Yes	Full length-IgG format, Fc fusions, scFv, VHH
Tethered variable CL or common LC bsAbs	None	2	VL-HC fusion (VLfH) or none	Short (Gly4Ser)_4_-linker or none	Intact BsAb generation in a single cell line by fusing the VL domain residues (1-R108) genetically to the antibody HC via (G_4_S)_4_ linker and CL co expression	co expression	None	ProteinA	yes if supernatant compatible	Not needed	Full-length-IgG format

## Abbreviations

aa	Amino acid
ADC	Antibody-Drug Conjugate
BLI	Biolayer interferometry
CL	Constant domain of a Light chain (of an antibody)
DARPin	Designed Ankyrin Repeat Proteins
Fab	Fragment antigen binding (of an antibody)
FAE	Fab-arm exchange
LC	(Antibody) light chain
LCMS	Liquid Chromatography-Mass Spectrometry
mTGase	Microbial Transglutaminase
MoA	Mode of Action
NSCLC	Non-Small Cell Lung Cancer
PTS	Protein Trans-Splicing
RPLC	Reverse Phase Liquid Chromatography
sdAb	Single domain Antibody
SrtA	Sortase A
TNF	Tumor Necrosis Factor
VHH	Variable Heavy chain domain of Heavy chain antibodies
